# Activation, incompatibility, and displacement of FIB replicons in *E. coli*

**DOI:** 10.1093/nar/gkaf275

**Published:** 2025-04-10

**Authors:** Georgina S Lloyd, Elton R Stephens, Alessandro Di Maio, Christopher M Thomas

**Affiliations:** School of Biosciences and Institute of Microbiology and Infection, University of Birmingham, Edgbaston, Birmingham B15 2TT, UK; School of Biosciences and Institute of Microbiology and Infection, University of Birmingham, Edgbaston, Birmingham B15 2TT, UK; School of Biosciences and Institute of Microbiology and Infection, University of Birmingham, Edgbaston, Birmingham B15 2TT, UK; School of Biosciences and Institute of Microbiology and Infection, University of Birmingham, Edgbaston, Birmingham B15 2TT, UK

## Abstract

Multi-replicon sex-factor F is the archetype of the largest plasmid group in clinical Enterobacteriaceae. Such plasmids spread antimicrobial resistance (AMR) and virulence functions in commensal bacteria of humans and animals. Displacing (curing) these plasmids by blocking replication and neutralizing addiction is successful with the curing cassette on a high-copy-number vector but, with conjugative IncP-1 plasmid RK2 as vector for our “anti-F cassette”, displacement of F’prolac is inefficient unless curing-plasmid copy-number is raised 1.5- to 2-fold. Here we report that it is the anti-FIB segment, originating from FIB-FII plasmid pO157, which needs potentiation. We show that the FIB replicon in F (F-FIB) is defective due to a sub-optimal *rep* ribosome-binding-site (rbs) but can be activated by FIB-Rep protein expressed from our anti-FIB segment joined to RK2. Deleting FIB-*rep* from the anti-F cassette removed the need for potentiation. A pO157-FIB single-replicon plasmid was displaced efficiently by the complete anti-F cassette without potentiation, but an F-FIB plasmid, mutated to have a pO157-like *rep* rbs, was not, indicating that sequence divergence between F and pO157 FIB replicons has weakened their negative cross-reactivity. Thus, raising vector copy-number slightly may be sufficient to increase displacement of plasmids similar but not identical to the sequences in the curing cassette.

## Graphical Abstract

**Graphical Abstract:**
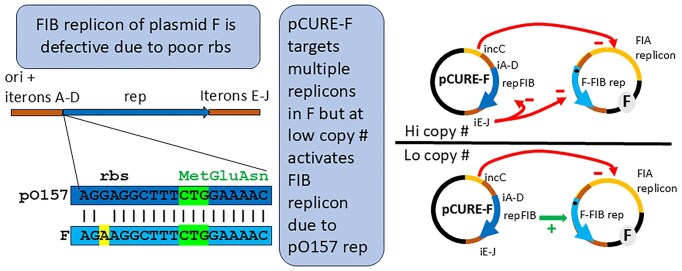

## Introduction

Bacterial plasmids are autonomous genetic elements that have evolved the ability to replicate in-step with the growth of their host. The first plasmid discovered was named F because it promoted fertility (gene exchange between bacteria) in *Escherichia coli* [1, 2]. This property has been essential for many of the most important discoveries in bacterial genetics, while the study of how F works as a plasmid, in terms of replication, stable inheritance, conjugative transfer, and carriage of transposable elements, has revealed many of the mechanisms and processes that underpin bacterial evolution [3]. Although some plasmids are successful without carrying genes that directly benefit their hosts, many plasmids do carry genes that encode an advantageous phenotype, antibiotic resistance being the most topical example [4]. Considerable effort is being directed towards finding ways to tackle the problems of antibiotic resistance [5], one of which is to explore how plasmids can be eliminated, removing all the resistance genes they carry in a single step [6].

**Figure 1.**
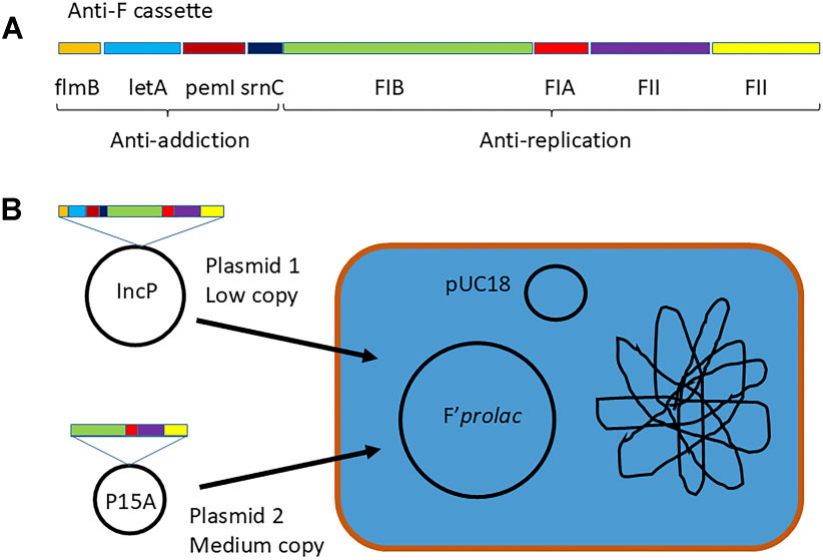
The anti-F cassette (**A**) and our strategy (**B**) to determine which segments need to be potentiated. The complete anti-F cassette was introduced on the low(ish) copy number Plasmid 1 (IncP-1 plasmid pCT549 with wild-type *oriV*) while sub-segments were introduced on a medium copy number Plasmid 2 (P15A replicon plasmids derived from pACYC184). The test strain was JM109 with pUC18 which complements the *lac* deletion in F’prolac to give Lac^+^ (blue) on Amp IPTG X-gal plates.

One strategy to displace plasmids is to use the plasmid's natural genetic functions to block replication and neutralize the addiction systems that normally prevent growth of plasmid-free segregants [7–9]. The essence of our general approach to achieve this plasmid displacement or “curing”, is to assemble a cassette of replication control and anti-addiction genes (Fig. 1A) which is joined to an unrelated vector [7]. When such a pCURE plasmid is introduced into a bacterium carrying the target plasmid, the latter is diluted out from the population due to lack of plasmid replication and survival of bacteria that have lost the plasmid [7].

Our initial pCURE plasmids were based on a high copy number vector with a pMB1 replicon [7]. The first anti-F cassette was designed to target pO157 from *E. coli* O157 [10], which was difficult to displace by other methods due to its possession of multiple replicons and addiction systems. Plasmid replicon typing, particularly with clinical and veterinary samples, over the last two decades has catalogued many such multi-replicon plasmids, of which the F-like plasmids are by far the largest group [11–13]. Our initial plasmid, pCURE1, targeted just the FIB and FII replicons of the specificity types in pO157 (Refseq NC_007414.1) but, to extend activity to other F-like plasmids, we added functions to block the FIA replicon of F (Refseq NC_002483.1) and additional FII (also called FIC) replicon types, generating pCURE2 [7].

To develop the potential applications of pCURE plasmids we determined whether the curing cassette would work when carried by a low copy number, conjugative broad-host-range IncP-1 plasmid as vector [9]. As our model target plasmid we used a derivative of F carrying part of the chromosome, F’prolac present in *E. coli* JM109 [14], allowing us to monitor plasmid displacement by loss of the Lac^+^ phenotype on X-gal/IPTG plates when pUC18 was also present in the bacteria. Although the FII replicon is not functional in F due to an IS insertion [15], plasmid F does carry functional FIA and FIB replicons [16]. We found that use of the wild-type RK2 plasmid as vector did not give efficient curing, but that deletion of a region adjacent to the RK2 replication origin that includes previously uncharacterized TrfA-binding sequence iteron 10, causing an 1.5- to 2-fold increase in copy number, gave a system that worked very well [9]. This need for potentiation was not confined to F-like plasmids, but also applied to IncK-plasmids [9].

To provide a rational basis for further development of the pCURE approach we needed to understand this potentiation and address any fundamental questions about plasmid replication control that this might raise. Studies on a variety of plasmids have shown that the negative feedback loop that shuts off replication as copy number rises can function in different ways [17]. In some cases, control is provided by repressors of transcription or translation which can be protein or RNA molecules [17] and in other cases by interaction of the replication origin with a second DNA segment, either another origin or a *cis-*acting inhibitory segment, mediated by a negatively-acting dimeric form of the plasmid's Rep protein, so called “handcuffing” [18]. Both strategies are observed in the replicons found in F-like plasmids. The FIA replicon uses handcuffing between the *oriV* and *incC* regions [19] while the FIB replicons have a very similar organization and probably do the same [20]. The FIC/FII replicon uses RNA and protein repressor feedback loops [21]. F-like plasmids are the most common carriers of antibiotic resistance genes in clinical isolates of Enterobacteriaceae [22]. Therefore, it was of both fundamental and applied interest to determine whether the potentiation by increased copy number was due to a general effect on all elements in our anti-F cassette or was specific to a subset of the curing functions. Our data implicate the FIB replicon in F as the basis for the anti-F cassette needing potentiation. They explain why the F FIB replicon is partially defective and provide a clearer framework for designing and exploiting plasmid curing cassettes.

## Materials and methods

### Bacterial strains, plasmids, and growth conditions

*Escherichia coli* strains used were C600 [23], C2110 [24]; DH5α [25], NEB5α (www.neb.com), JM109 [26], Nissle 1917 [27], and Sakai [10]. Plasmids used in this work are listed in Supplementary Table S1. Bacteria were cultured aerobically, in either L-broth (LB)/L-agar (LBA) [28], or M9 Minimal Medium [29] (supplemented with amino acids at 50 μg ml^−1^) at 37°C. Final antibiotic concentrations were: ampicillin (Ap or Amp), 100 μg ml^−1^; kanamycin (Km or Kan), 50 μg ml^−1^; chloramphenicol (Cm or Cam), 50 μg ml^−1^; nalidixic acid (Nal), 25 μg ml^−1^; rifampicin (Rif) 100 μg ml^−1^; and tetracycline (Tc or Tet), 25 μg ml^−1^. For the blue/white screening, L-agar was supplemented with X-gal (20 μg ml^−1^) and IPTG (0.5 mM).

### DNA analysis and manipulation

Restriction enzymes and T4 DNA ligase were purchased from New England Biolabs (NEB); Taq DNA polymerase was from Invitrogen; Velocity proof-reading DNA polymerase was from Bioline; Q5 High-Fidelity Taq polymerase was from NEB. Polymerase chain reaction (PCR) amplification of DNA used the oligonucleotide primers from Merck/Sigma−Aldrich listed in Supplementary Table S2, which is subdivided into sections with headings that indicate the purpose of the primers. PCR reactions were cycled in a SensoQuest Lab Cycler following standard procedures [30]. PCR products were purified using the Illustra GFX^TM^ PCR DNA and Gel Band Purification Kit (GE^TM^ Healthcare). PCR products generated with appropriate primers were joined by Spliced Overlap Extension (SOE) [31] starting with four PCR cycles before primers were added to amplify the SOE’d product. Site-directed mutagenesis was carried out with a Q5 SDM kit from NEB incorporating a KLD (Kinase-Ligase-DpnI) step followed by transformation of super-competent *E. coli* NEB5α. Plasmid DNA was isolated using the Plasmid MiniPrep DNA Extraction Kit (Bioline) adapted from the alkaline lysis method of Birnboim and Doly [32]. DNA sequencing reactions were prepared and run on an ABI 3730 DNA analyser (Functional Genomics Facility, University of Birmingham, U.K.) following the chain termination method [33]. Other standard procedures were carried out as described in the Laboratory Manual of Sambrook and Russell [30].

### Construction and manipulation of plasmids

The anti-F replication functions from pCURE2 were amplified as a block, and as separate segments, with primers listed in Supplementary Table S2, which allowed insertion into pLAZ2 as BglII-EcoRI fragments. The complete FIB (from pO157 and F’prolac) and FII (from pO157) replicons were also amplified and inserted into pLAZ2 as BglII-EcoRI fragments but the FIA replicon from F contains a BglII site so it was inserted as an EcoRI-PacI fragment. Point mutations in the F FIB replicon to change amino acids I234 and F281 were introduced using a Q5 SDM kit (NEB). The hybrid FIB replicons were made by amplifying F and pO157 segments with inner primers that would allow joining by SOE. The changes to the start codon in both F and pO157 FIB replicons and the introduction of the CAC > CAT suppressor mutation into the pLAZ_F-FIB_GTG plasmid were also done by SOE.

The pLAZ_F-FIB_egfp plasmid was constructed by SOE to clone the *egfp* gene immediately after the F-FIB *rep* gene (and therefore under the control of the F-FIB promoter) and before the downstream F-FIB iterons. The F-FIB *rep* gene was amplified from F’prolac with primers to create an EcoRI site and an overlap with the start of the *egfp* gene, and the F-FIB downstream iterons were amplified from pLAZ_F-FIB with primers creating an overlap with an LAA instability tag [34] included in place of the stop codon for the *egfp* gene and a SalI site. The *egfp* gene was amplified from pCT::egfp with two primers that created the overlaps for SOE with the F-FIB *rep* gene and the F-FIB downstream iterons. Following SOE, the purified PCR product and pLAZ1 were cut with EcoRI and SalI followed by ligation generating pLAZ_F-FIB_egfp.

To construct the FIB-FII dual replicon plasmids we started from the pLAZ_OFII plasmid that contains three key unique restriction sites: EcoRI and SalI that define the FII replicon and NheI that can generate an EcoRI-NheI fragment that includes both the FII replicon and the *cat* gene. The F-FIB replicon was amplified intact or in two pieces with mutagenic primers to change the rbs to be the same as in pO157 FIB. The Wild Type (WT) and mutant FIB replicons were amplified with primers to create an EcoRI-NheI fragment and also with alternative primers to generate an overlap with and allow SOE to join it with an *aph* gene defined at the other end by a SalI site. Cutting relevant amplicons and pLAZ_OFII with EcoRI and SalI followed by ligation generated Km^R^ dual replicons while doing the same with EcoRI and NheI generated Cm^R^ dual replicons.

Making derivatives of the anti-F cassette in pCT549 was done as described previously [9] after first changing the antiF cassette in pLAZ3 that has the anti-F as part of a BglII-antiF-PacI-*oriV*-EcoRI segment. The derivatives were then inserted into pCT549 by BglII cutting, ligation, and transformation into C2110. After plasmid DNA isolation to identify plasmids with correct orientation, resolution by EcoRI digestion and ligation gave the final product. Long range SDM PCR was carried out with Q5 High-Fidelity Taq polymerase using the NEB-recommended primer design and conditions.

### Comparison of plasmid copy number

For relative copy number comparison, at least triplicate selective overnight (16 h) cultures of *E. coli*, carrying both an internal standard plasmid and the query plasmids, were grown at 37°C in LB plus appropriate antibiotics, to select for both plasmids, with shaking at 200 rpm [35]. Plasmid DNA was extracted and then linearized with EcoRI to give a single fragment per plasmid that binds ethidium bromide more uniformly. After agarose gel electrophoresis, band intensities were determined with Biorad Imagelab 5.2 Software and normalized against the internal standard. Colonies from serial dilution of the cultures were replica plated to determine % plasmid carriage and plasmid copy number adjusted if there was evidence of significant plasmid loss.

### Testing curing efficiency

Test plasmids were introduced into target bacteria by transformation. Displacement of F’prolac from JM109 (pUC18) was determined by growth on L-agar with IPTG and X-gal when the target strain additionally carried pUC18 (selected for with Amp) to give a Lac^+^ phenotype (blue colonies) due to the presence of the LacZα–fragment encoded by pUC18, so that loss of F’prolac gave a Lac^−^ phenotype (white colonies) [9]. It carries the same F’prolac as TG1 (a JM101 derivative) whose sequence (NZ_CP128219.1) is 100% identical to the *E. coli* K12 F plasmid sequence (NC_002483.1). Loss of antibiotic resistance plasmids was determined either by initial selection for only the incoming plasmid and replica plating onto fresh plates to estimate resident plasmid retention, or by spreading the transformation mix on agar with selection for the incoming plasmid as well as with and without selection for the plasmid to be displaced.

## Results

### The anti-FIB locus in the anti-F curing cassette needs to be potentiated

**Figure 2.**
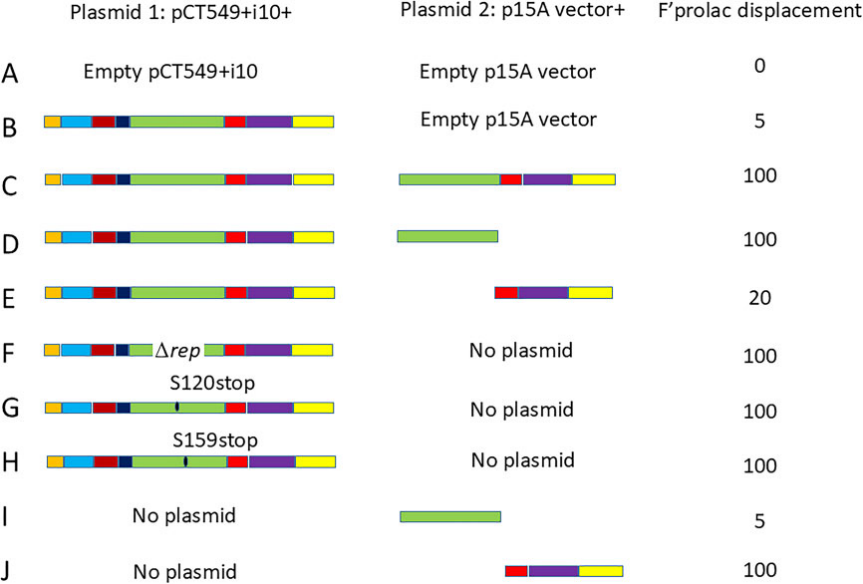
Segments needed to potentiate the anti-F cassette (colour-coded as in Fig. 1A). As in Fig. 1B, displacement was tested by co-transforming *E. coli* JM109 (pUC18) with Plasmid 1 plus anti-F cassette and Plasmid 2 plus sub-segments (control lines A and B; tests lines C–E). Plasmid 1 derivatives constructed to test the effect of inactivating the FIB *rep* gene were introduced alone (lines F–H). The effect of sub-segments in Plasmid 2 were tested alone (line I–J). Displacement is measured by the % white colonies appearing on Tc Cm IPTG Xgal plates.

To determine which part(s) of the anti-F cassette needs potentiation by raising copy number we used a two-plasmid approach as illustrated in Fig. 1. This involved taking the whole anti-F cassette joined to the low copy number IncP replicon with full copy number control functions (pCT549 + i10, Plasmid 1) and subsets of the anti-F cassette joined to the medium copy number P15A replicon (pLAZ1, Plasmid 2). Curing was tested by double transformation of both plasmids into JM109 that carries F’prolac and pUC18 as illustrated in Fig. 1B. To favour double transformant bacteria for screening, we added antibiotics to select for both incoming plasmids after 1 h expression period and incubated for a further 3 h before spreading on selective plates to determine loss of the F’prolac. This showed that, although the unpotentiated anti-F cassette (present only on Plasmid 1) does not stop F’prolac from replicating completely, it does cause a detectable increase in segregational loss of F’prolac per generation, suggesting some effect on replication and therefore copy number (Fig. 2, line B). This is indicated by ∼5% of colonies being white (Fig. 2, line B) as well as the observation that the blue colonies with F’prolac were not as intensely blue as the controls and, when allowed to grow enough, had a white rim (Supplementary Fig. S1). However, adding just the anti-F *rep* functions on Plasmid 2 (Fig. 1B) gave complete displacement within the detection limits of the experiment (Fig. 2, line C), showing that the anti-addiction functions do not need to be potentiated. Further sub-cloning then showed that having just the anti-FIB segment in plasmid 2 is sufficient to give complete displacement (Fig. 2, line D), while the remaining anti-replication functions as a block (anti-FIA and anti-FII) are not sufficient (Fig. 2, line E).

### Inactivation of the FIB *rep* ORF removes the need for potentiation

**Figure 3.**
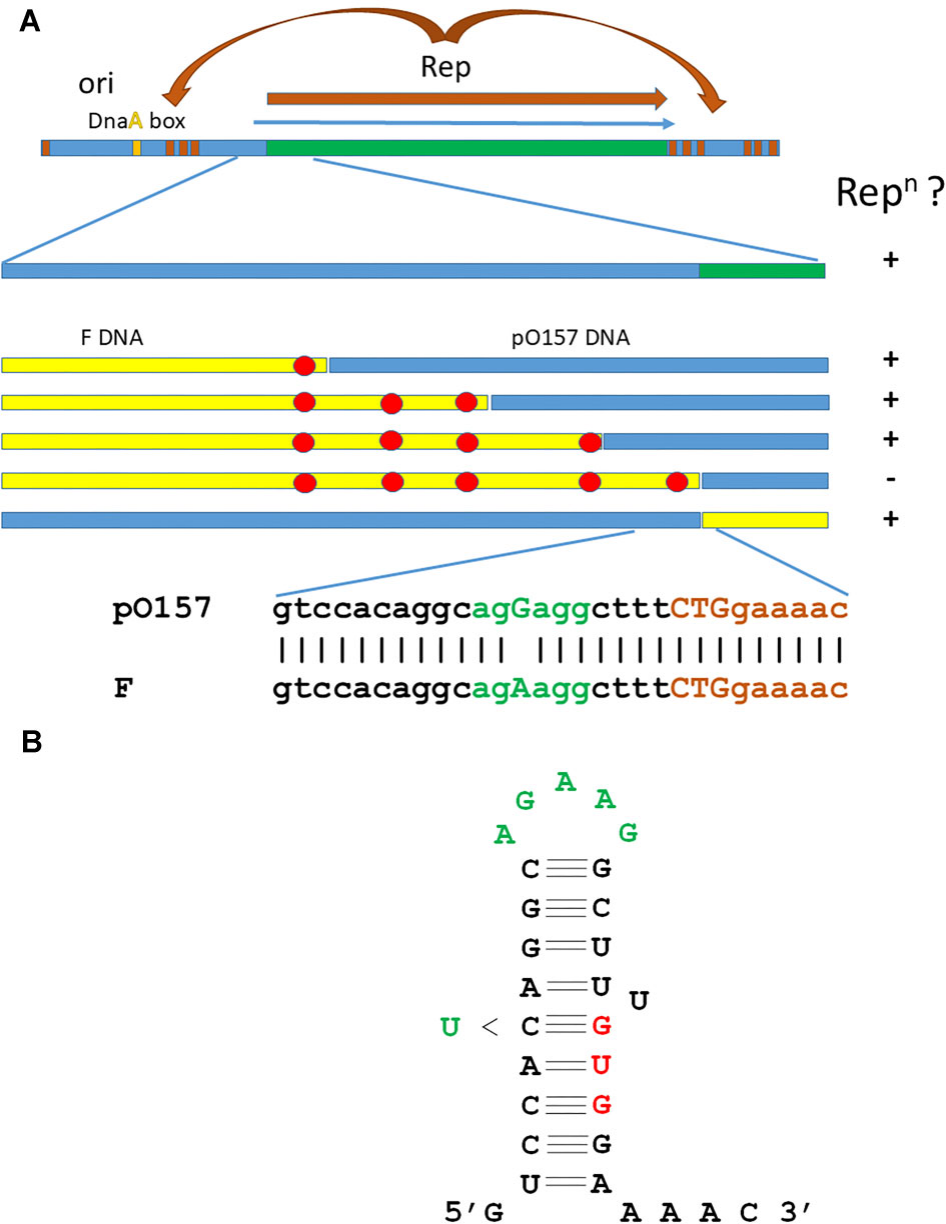
(**A**). Hybrid F/pO157 FIB replicon analysis. Structure of the FIB replicon is shown at the top. The *rep* gene is shown flanked by iterons which bind Rep as well as the DnaA binding site. Dual replicon plasmids with P15A and hybrid FIB replicons (pLAZFIB HybridA to H as shown in Supplementary Table S1) were used to map the sequence change in F plasmid DNA resulting in reduced ability (−) for replication (Rep^n^) in *E. coli* C2110. The discs represent nucleotide differences between F and pO157 DNA. (**B**). The predicted secondary structure resulting from CTG > GTG as start codon mutation, also showing the C to T mutation that restores gene function.

We were not sure at the time of designing the original pCURE [7] which FIB segments would be most effective and so took a large proportion of the replicon, including both upstream and downstream iterons with the intervening *rep* gene but not the putative *ori* region (Fig. 3 and Supplementary Fig. S2) [36, 37]. The anti-FIB segment in the anti-F cassette is therefore different from the anti-FIA and anti-FII replicon segments in the fact that it provides the Rep protein that the replicon needs to function. We therefore hypothesized that the presence of the FIB *rep* gene may be a reason why the activity of the FIB segment needs to be potentiated. To test this hypothesis, we inactivated the FIB *rep* orf in the anti-F cassette in three different ways - with an in-frame deletion (pCT549 + i10 + aF ΔFIBrep) and with two different point-mutations resulting in stop codons at positions 120 or 159 and determined whether these mutations were each sufficient to allow curing with the anti-F cassette joined to the WT IncP-1 replicon (pCT549 + i10). As predicted, each of these mutants alone allowed the anti-F cassette to cause efficient displacement of F’prolac without copy number elevation (Fig. 2, lines F–H). Thus, it appears that the FIB Rep protein encoded by the anti-F cassette is the reason why the original anti-F cassette needs to be potentiated.

### The FIB replicon is partially defective in F

We also checked the effect of the anti-FIB segment alone and combined anti-FIA plus anti-FII functions on stability of the F’prolac in the absence of pCT549 + i10 + aF. Surprisingly the anti-FIA and FII segments together were sufficient to block replication, although they were not able to neutralize activation of the addiction systems (so transformant colonies were very small), while the FIB segment alone had no apparent effect on F’prolac maintenance (Fig. 2, lines I and J). Since the FII replicon in F is inactive due to a transposon insertion [15], this suggested that the FIB replicon alone is not able to maintain F’prolac when the FIA replicon is blocked. This may be consistent with the FIB replicon being unstable at faster growth rates (mean generation times, mgt, <60 min [16].

These observations suggest the hypothesis that the F’prolac FIB-*rep* gene is at least partially defective and that this defect can be complemented by *rep* in the anti-FIB segment in the anti-F cassette in pCT549 + i10 + aF since it comes from pO157 (from the *E. coli* Sakai O157 strain) rather than F. We therefore amplified the FIB replicons from F’prolac and pO157, joined them to the PolI-dependent P15A plasmid pLAZ2 (yielding pLAZ_F-FIB and pLAZ_O-FIB respectively) and tested functionality by transforming *E. coli* C2110 in which the P15A replicon is non-functional due to the PolA1 allele. Using LB-agar (plus appropriate antibiotics) the O-FIB plasmid transformed C2110 efficiently while the F-FIB plasmid gave no colonies unless pCT549 + i10 + aF (with iteron 10) was present, when hundreds of small colonies were obtained (Table 1) consistent with the anti-F cassette activating the F-FIB replicon. Only about 50 tiny colonies were obtained with the F-FIB when C2110 contained pCT549-i10 + aF (without iteron 10 and thus higher copy number) as expected from the shift in balance of positive and negative functions when copy number is raised. These results indicate that the F-FIB replicon is not functional in C2110 in L broth unless activated by the anti-F cassette at low copy number.

**Table 1.** Ability of FIB replicons from pO157 and F to transform *E. coli* PolA^−^ strain C2110

		Transformation efficiency^a^	
Agar medium	Plasmid present in C2110	Parental plasmid pO157	Parental plasmid F
LB	None	+++	−
LB	pCT549 + i10 + aF	++	+++
M9glucose + his	None	+++	++
LB	pCT549 + i10 + aFΔFiBrep	−	−
LB	pCT549-i10 + aF	(+)	++
LB	pCT549-i10-aF	+++	ND^b^

^a^+++, many (>100) healthy transformants; ++, reduced number (<50) of slower growing transformants; (+), occasional transformants that may be recombinants; −, no transformants.

^b^ ND, Not done because transformants were obtained with pCT549-i10 + aF present.

We also selected transformants in C2110 on M9 medium and obtained hundreds of transformants with pLAZ_O-FIB but only about 50 with pLAZ_F-FIB (Table 1). Confirmation that the transformants contained the correct plasmids was obtained by agarose gel electrophoresis of plasmid DNA extracted from saturated liquid cultures grown in M9 medium from both sets of transformants. Transformation back into DH5α gave ∼5-fold more transformants for pLAZ_O-FIB compared to pLAZ_F-FIB, which was consistent with the intensity gel bands for pLAZ_O-FIB being about five times stronger than those of pLAZ_F-FIB as determined by Quantity One image analysis (data not shown). DNA sequencing confirmed that there were no changes in the F-FIB replicon. These results confirmed the F-FIB defectiveness relative to the O-FIB and functionality dependent on growth medium.

We considered the possibility that the PolA1 defect might disproportionately affect the poorly functioning F-FIB replicon and so used an NheI-XbaI digestion and ligation to delete the P15A replicon from the pLAZ derivatives described above and transformed DH5α. The pO157-FIB replicon on its own was easily obtained in this way, selecting transformants on either LB or M9 agar, but the F-FIB replicon was obtained only with M9 agar at 30°C and transformant colonies were small and slow-growing (data not shown). The plasmid was rapidly lost if antibiotic selection was removed, consistent with the observations of Lane and Gardner [16].

### The F FIB replicon defect is due to *rep* having a weak ribosome binding site

Sequence comparison showed two amino acid differences between the Rep proteins of F and pO157 at positions 234 and 281 (Supplementary Fig. S3) which may be responsible for the observed replication defect. To determine whether this is the case, we mutated each amino acid separately in the F-derived replicon (I234V and F281L) and then together to see if this would reactivate the replicon. Neither the single mutant nor the double mutant reactivated the replicon (data not shown), indicating that it is not the protein that lacks function. Further comparisons showed several single base pair differences upstream of the *rep* orf, which unusually starts at a CTG codon [36, 38] that may affect transcription or translation of the *rep* gene. We therefore created a series of hybrid replicons using F upstream sequences and pO157 downstream sequences to identify which sequence difference(s) is responsible for the loss of replication function by transformation of the PolA^−^ strain C2110 (Fig. 3A). The results showed that replication ability was only lost when the difference closest to the start of the *rep* gene was incorporated, creating a poorer rbs (Fig. 3A). We then created additional hybrid replicons with the pO157 upstream sequences and the F *rep* orf and this showed that the rbs single nucleotide polymorphism (SNP) was sufficient to cause loss of replication ability in C2110 (Fig. 3A).

We searched specifically with the DNA sequence around the start of the FIB *rep* gene and found that all F plasmids that have been sequenced have this poorer ribosome binding site, suggesting that this property is likely to have been present in the ancestral F plasmid. In addition, it occurs in resistance plasmid R386 (L01254.1) conferring tetracycline resistance [39] and pHH502 (L01255.1) conferring resistance to trimethoprim and mercuric ions [40]. Neither of these plasmids is completely sequenced but their FIB replicons have been cloned and sequenced [38]. The FIB sequence from pHH502 has only one mismatch from the F sequence while R386 has quite a few differences, particularly around the start and the end of the *rep* gene. Surprisingly pHH502 has an IncI replicon that can be lost by a specific recombination event leaving a plasmid with just IncP-1 incompatibility, according to Nugent *et al.* [40] unless the FIB replicon was missed due to its poor functionality. We also identified a different clade of FIB replicons with a SNP (highlighted in cyan) in this region (GTCCACAGGCTGGAGGCTTTCTGGAAAAC) but this clearly does not have a major effect on the rbs function because the clade includes P307, the source of the FIB replicon studied by the Bergquist lab thirty years ago [37].

### Suppression of the F FIB replication defect

An interesting feature of the FIB *rep* gene is the fact that it uses CTG as the start codon [37, 39]. To test the importance of the CTG start codon in setting the expression of *rep* at an appropriately low level, we mutated this codon to the different possible codons (TTG, GTG, and ATG, in order of increasing frequency of usage) in both the pO157- and F-FIB replicons in the FIB-P15A dual replicon plasmids. We also mutated the CTG to CTC (which is a synonymous codon) in the pO157-FIB replicon just to check that it is the start codon and showed that these derivatives could not replicate in C2110 (Supplementary Table S3). We found that the ATG and TTG substitutions made the F-FIB replicon functional in C2110 and did not harm the pO157 FIB replicon (we had considered the possibility that raising the *rep* gene expression might have a negative effect) (Supplementary Table S3). However, to our surprise the GTG substitution did not activate the F FIB replicon and inactivated the pO157 FIB replicon instead. Examination of the leader messenger RNA (mRNA) sequence suggested this may be due to formation of a hairpin (TCCA**C**AGG….TTT**GTG**GA) facilitated by the C > G mutation (Fig. 3B). We therefore mutated the complementary C in the upstream sequence in the F FIB replicon to T (CAC > CAT) and found that it could now replicate. This confirms the use of the CTG start codon and suggests that it is selected to give just enough expression, when combined with a good rbs, to allow replication.

With the idea of monitoring expression of the F-FIB *rep* operon in LB and M9 medium, we inserted an *e-gfp* reporter gene (modified to destabilize the fluorescent protein) downstream of *rep* as a transcriptional fusion within otherwise complete F-FIB and O-FIB replicons in pLAZ_F-FIB and pLAZ_O-FIB respectively. We were able to detect fluorescence in both types of media with the majority of cells giving a weak signal and a minority a stronger signal (Supplementary Fig. S4). Surprisingly, we found that both F- and O- derivatives were now able to transform C2110 efficiently in both media, indicating that the Rep deficiency in the F-FIB replicon had been overcome. Since there are no obvious potential secondary structures in the region downstream of the *rep* ORF that could protect the mRNA from 3′ exonuclease degradation, it may be that the presence of the *e-gfp* gene increases stability of the *rep* transcript and therefore elevates the production of Rep. This could suggest that the FIB *rep* mRNA normally has a very short half-life to link Rep production tightly to *rep* transcription.

### Curing cassette activity against individual replicons

To determine the ability of the anti-F cassette to work against individual replicons we inserted each replicon separately, in the absence of an active partitioning system, into pLAZ2 and then deleted the P15A replicon as described in the ‘Materials and methods’ section. The FIA replicon was taken from F’prolac while FIB and FII were taken from pO157. We first determined the relative copy number of these single replicon plasmids. Each single replicon plasmid (FIA, FIB and FII) was introduced into *E. coli* carrying the compatible IncP-1 vectors pCT549 with and without iteron 10 which were used as internal controls. DNA was extracted and relative fluorescence determined. The relative copy numbers of the FIA and FII single-replicon plasmids are similar to each other but significantly lower than the FIB replicon (Table 2 and Supplementary Table S4). The data also confirm that the removal of iteron 10 from the *oriV* region for the IncP-1 replicons causes an increase in copy number. Perhaps most significantly, the IncP-1 replicon with iteron 10 has a copy number lower than the isolated FIB replicon while the mutant IncP-1 replicon (pCT549-i10) lacking iteron 10 has a copy number higher than that of any of the F replicons, which correlates with curing efficiency.

**Table 2.** Relative copy numbers of F replicon plasmids compared to IncP plasmids based on relative fluorescence intensity per kb in agarose gels shown in Supplementary Table S4 and Supplementary Figs S5 and S6

	Versus IncP-1 replicon pCT549 + i10			Versus IncP-1 replicon pCT549-i10		
	Mean of ratios^a^	SD	p^b^	Mean of ratios^a^	SD	p^b^
FIA	0.829	0.030	FIAvFIB = 2 × 10^–7^	0.492	0.043	FIAvFIB = 2.6 × 10^–7^
FIB	1.132	0.057	FIBvFII = 9.5 × 10^–5^	0.775	0.044	FIBvFII = 2.7 × 10^–6^
FII	0.777	0.024	FIAvFII = 0.068	0.456	0.078	FIAvFII = 0.169

^a^Mean of 6 ratios per replicon.

^b^ *P*-value from comparison of means by TTest calculated in Excel.

To check that it is only the anti-FIB function in our anti-F cassette that needs to be potentiated, we tested pCT549 + i10 + aF (pCT549++) and pCT549–i10 + aF (pCT549−+) against the individual FIA and FII replicons from F and pO157 respectively as described in the ‘Materials and methods’ section. Interestingly, the FIA replicon (in the absence of an active partitioning system) seemed to be significantly unstable as one might expect for random segregation of a low copy number plasmid and, while the anti-F cassette did cause very efficient displacement, it did not appear to block replication completely when provided from pCT549 + i10 as evidenced by the appearance of small colonies on the double selection transformation plates. By contrast the FII replicon from pO157 appeared to be very stable but disappeared completely when blocked by the anti-FII elements in the cassette (Table 3).

**Table 3.** Displacement of single replicon plasmids by the anti-F cassette and derivatives

	Displacement of single replicon plasmids from *E. coli* C2110^a^			
Test plasmid	pO157 FIB	F FIB Mut^b^	F FIA	pO157 FII
pCT549 + i10^c^	-	-	-	-
pCT549 + i10 + aF^d^	+	(+)	(+) 50%–100%	+
pCT549 -i10 + aF^e^	+	+	(+) 50%	+
pCT549++ΔFIB*rep*^f^	+	+	ND^i^	ND^i^
FIB upstream^g^	-	ND^i^	ND^i^	ND^i^
FIB downstream^h^	+	ND^i^	ND^i^	ND^i^

^a^−, no displacement observed; +, displacement observed.

^b^This replicon has a mutated *rep* rbs sequence like that in pO157 making the FIB replicon functional.

^c^Also called pCT549+−, i.e. IncP replicon with iteron 10 but without the anti-F cassette.

^d^Also called pCT549++, i.e. IncP replicon with iteron 10 and with the anti-F cassette.

^e^Also called pCT549−+, i.e. IncP replicon without iteron 10 but with the anti-F cassette.

^f^pCT549 + i10 + aF but FIB *rep* gene deleted leaving FIB iterons intact.

^g^The upstream region with iterons BCDD’ is associated with the replication origin.

^h^The downstream region with iterons E to K is associated with replication control.

^i^Not determined.

For the pO157 FIB replicon it was interesting that both pCT549++ and pCT549−+ showed efficient displacement whereas only pCT549−+ showed efficient displacement against F’prolac. To test whether the poor displacement of F’prolac is due to the fact that it produces little FIB Rep itself, we created an FIB single replicon plasmid by mutating the rbs in the F-FIB replicon so that it was the same as the pO157 rbs and tested the sensitivity of this to displacement (Table 3). This showed that this F-FIB replicon is less easily displaced than the pO157 FIB replicon despite producing more normal levels of Rep. Since we had already created hybrid replicons that include the fully functional rbs, we tested these in C2110 (to inactivate the P15A replicon) for displacement by pCT549++ and pCT549−+. The results showed that hybrids A, B, and C, which include the F-FIB ori region, appeared less susceptible while hybrid G, which includes the pO157-FIB ori region, is more susceptible (Fig. 3). This suggests that it is the divergence in the *ori* region that is responsible for the differences in sensitivity to displacement.

**Figure 4.**
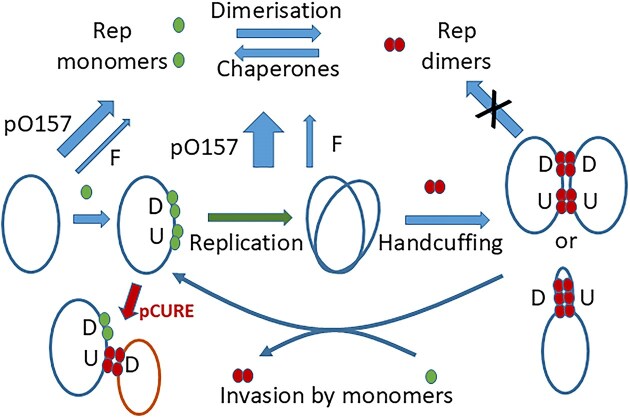
Scheme for replication cycle of the FIB replicon and action of pCURE. Expression of Rep is weak and autoregulated by Rep-iteron interaction. Handcuffing mediated by Rep dimers at upstream (U) and downstream (D) iterons can occur both inter- and intra-molecularly. The handcuffed replicon remains inactive until cell growth dilutes Rep and enough monomer accumulates to invade and disrupt the DNA–Rep sandwich. The downstream iterons in pCURE must act by interaction with the upstream iterons in the target plasmid.

The FIB replicon has two sets of Rep protein binding sites (Iterons), upstream and downstream of *rep*, that are predicted to be involved in handcuffing to control replication and incompatibility (Fig. 4 and Supplementary Fig. S2). To clarify the requirements for efficient FIB replicon displacement we separated the two sets of iterons joined to the P15A replicon from pACYC184 and tested for the ability to displace the FIB replicon plasmid. The results showed that the EFGHIJ block of iterons alone was sufficient for efficient curing of the pO157 replicon, while the BCDD’D iteron block alone did not cause displacement (Table 3). This strongly points towards a model in which the downstream iterons target the upstream region either in *cis* or in *trans*.

### Competition between dual replicon plasmids

The results above raise the question whether incompatibility between two FIB replicons would be reduced if only one of them produces “normal” levels of Rep protein and whether incompatibility would be symmetric or asymmetric (ie one of the two plasmids is preferentially lost). We therefore created two sets of dual replicon plasmids with the FII replicon and either F-FIB WT (i.e. partially defective due to the rbs SNP) or F-FIB MUT (with functional rbs) replicons joined to either the *cat* or the *aph* selectable markers. They were first tested for displacement by our anti-F cassette and as expected both WT and MUT were poorly displaced by pCT549++ but efficiently displaced by pCT549−+. Both WT and MUT were then tested for displacement by the WT plasmid with the alternative selectable marker (Supplementary Table S5). No consistent difference was seen in the sensitivity of the WT or mutant FIB replicons to displacement.

## Discussion

This project set out to explain why our anti-F cassette needs to be potentiated when attached to a low copy number conjugative plasmid [9], but it has also revealed a considerable amount of new information about the FIB replicon.

A fundamental issue that we noted is that most FIB replicons annotated in Genbank accessions incorrectly show the *rep* start codon as a GTG, 14 codons downstream of the CTG start codon shown in Supplementary Fig. S2. The CTG was first correctly identified based on N-terminal analysis of the P307-FIB Rep protein published >30 years ago [38]. The current confused situation may have arisen because a subsequent paper from the same group [36] showed the downstream GTG as the start codon in their Fig. 3, comparing FIB DNA sequences from 10 different F-like plasmids. It is therefore important that our mutation of the pO157-FIB CTG start codon to synonymous codon CTC resulted in complete loss of replicon function, while our mutational analysis of the F-FIB *rep* start codon and rbs confirmed the role of the CTG. This data, from three different FIB replicons, thus allows us to argue that this CTG codon is most likely to be used in all the closely related FIB replicons and therefore should be the default position for annotation. The use of a CTG start codon is much rarer than TTG, the least frequent of the “normal” three (ATG, GTG, TTG) start codons, and in a recent analysis of nearly 85,000 genes in model bacterial genomes, only 20 genes (0.024%) were annotated as using CTG [41]. Given that the FIB *rep* is generally incorrectly annotated, it seems possible that there will be other instances of rare start codon usage being overlooked during whole genome analysis and annotation, even though the bioinformatic tools for high quality genome annotation do already exist [42].

Hecht *et al.* [41] showed that CTG gives about 10× less expression of their reporter gene than TTG, the poorest of the three common start codons (ATG > GTG > TTG). Perhaps significantly, the system they used involved an excellent rbs (5′ AGGAGA 3′) separated from the test codon by an ideal 7 nucleotides (TAAATAC). In line with this, the FIB CTG seems to be sufficient when combined with a good rbs (5′ GGAGG 3′) as in pO157 or insertion of an extra cds (gfp) downstream of the *rep* ORF that may stabilize the transcript when the rbs is less strong (5′ GGAAG 3′). The implication is that a small amount of FIB Rep is sufficient to drive efficient replication. By contrast, the *rep* gene from the formally very similar FIA replicon starts with an ATG but has only a moderately good rbs (5′ **GGA**tctgtcATG 3′ from F plasmid sequence accession AP001918). The fact that our results show that the FIB replicon remains functional when translation of the mRNA is increased by substitution of more efficient start codons indicates that the low level of Rep is not essential to stop the negative role of Rep (handcuffing) from dominating, nor to limit the amount of replication that can take place, since the copy number of such mutant plasmids is not elevated. It seems likely therefore, that the level is set to minimize the fitness-cost arising from RepA itself on the host while retaining replicon function, especially since the replicon seems most functional at slow growth rates [16]. On the other hand, since the copy number of the isolated FIB replicon appears to be higher than the isolated FIA or FII replicons (Table 2) it may be that when all three are present together, inactivation of the FIB replicon allows reduced copy number and thus reduces overall metabolic load on the host cell.

The fact that it was easier to displace the pO157-FIB plasmid than the F-FIB plasmid also made us wonder whether the rbs mutation for the F-FIB *rep* gene creates a potentially active replicon that has a competitive advantage in the presence of a plasmid that provides FIB Rep protein. In other words, has F acquired a “cheater” mutation [43], meaning that it no longer wastes energy on the FIB replicon because it has acquired the similar FIA replicon that is linked to partitioning and addiction functions but retains enough function, including the downstream iterons, to allow it to compete effectively when necessary. To test this, we created dual replicon F-FIB + FII plasmids, differing only in their selectable marker and their FIB *rep* rbs alleles and conducted classic incompatibility tests. However, we were unable to detect an advantage or a disadvantage for the plasmid with the weaker rbs. This may suggest that the F-FIB replicon's slightly greater resistance to displacement than the pO157-FIB replicon may be due to the small amount of sequence divergence (3% across the replicon; see Supplementary Fig. S2) between the two plasmids rather than because of carrying the less active rbs. We therefore compared single replicon FIB plasmids—WT pO157-FIB and Mutant F-FIB with the same rbs–start codon region sequence—and this confirmed that the F-FIB is less susceptible to displacement by pCT549++.

To establish which part of the F-FIB differences are responsible for the loss of sensitivity, we exploited the hybrids created while mapping the region responsible for the replication defect (Fig. 3). The results clearly indicated that it is the divergence in the *ori* region upstream of *rep* that results in reduced sensitivity. By contrast, our dissection of the FIB replicon showed that it is the downstream region that exerts the strongest inhibition of replication on a target plasmid. This is consistent with it being the direct interaction between Rep bound at the upstream (*oriV*) iterons in the target and downstream FIB iterons in our anti-F cassette that is responsible for the curing effect. It is also consistent with the model for FIA replication control [19] involving an intra-molecular sandwich mediated by Rep between the two sets of iterons as indicated in Fig. 4.

These results therefore underpin our conclusions about design of a plasmid-curing cassette to block replication of any plasmid, but particularly a multi-replicon target plasmid where it is not immediately clear whether all replicons identified from the DNA sequence are fully active. First, avoid including any coding region for a trans-acting function that can play a positive role in replication even if it might also have a negative role that one would expect to counter-balance the positive role. Second, with iteron plasmids it is generally better to include only the controlling iterons (in both FIA [19] and FIB (this work) replicons these are the downstream iterons) rather than the activating iterons associated with the origin region itself. Third, even minor sequence differences may affect replicon function and incompatibility which may alter curing ability unpredictably. Fourth, using a vector with slightly raised copy number can compensate for the weakened effectiveness of a slightly divergent set of curing functions. In this respect the difference between RK2 and pUB307 seems very convenient because it has a major effect on plasmid curing but a minor change in copy number [9].

## Supplementary Material

gkaf275_Supplemental_File

## Data Availability

All data is shown either in figures or tables in the published paper or in Supplementary data which are available at NAR online. The gel images in Supplementary Figures 3 and 4 are exported .tiff files from the .1sc files generated and analysed in Biorad Imagelab 5.2 Software which are available from the corresponding author on request.
