# Supplementary material for: Activation, incompatibility, and displacement of FIB replicons in E. coli
Source: Nucleic Acids Res. 2025 Apr 10;53(7):gkaf275. doi: 10.1093/nar/gkaf275 (PMC11983097; doi:10.1093/nar/gkaf275)
Supplement: gkaf275_Supplemental_File [file gkaf275_supplemental_file.pdf]

Supplementary Table S1. Plasmids used and constructed during this work.

Plasmid	Properties	Reference/Source
F'*prolac*	RepFIA RepFIB RepFIC/FIIA Tra^- in JM109 (Refseq accession: NZ_CP128219.1) PTU-FE[a]	Yanisch-Perron et al., 1985
pO157	RepFIB RepFIIA; Tra^-; (Refseq accession: NZ_AP018692.1) PTU-E5[a]	Burland et al., 1998; Makino et al., 1998
pCT::egfp	IncK; Km^R (Refseq accession: NC_014477.1) PTU-B/O/K/Z[a]	Cottell et al., 2014
pACYC184	P15A replicon, Cm^R Tc^R	Chang and Cohen 1978
pDS3	P15A replicon, Cm^R	Thomas, 1981
pCURE2	pMB1 replicon, *oriT*RK2, *sacB*, anti-IncF; Amp^R Kan^R	Hale et al., 2010
pLAZ1	pAYCYC184 derivative with sacB but EcoRI site in *cat* removed by site directed mutagenesis; Cm^R. tetA disrupted by homology arms for insertion into pUB307.	Lazdins et al., 2020
pLAZ2	pLAZ1 with anti-F cassette inserted as a BglII-AatII fragment; Cm^R	Lazdins et al., 2020
pLAZ3	pLAZ2 with *sacB* replaced by oriV of RK2 inserted as an XbaI-EcoRI fragment; Cm^R	This work
pLAZ_aFIB/FIA/FII	pLAZ2 with complete anti-F segment from pCURE2 inserted as BglII-EcoRI fragment; Cm^R	This work
pLAZ_aFIA-FII	pLAZ2 with anti-FIA/FII segment from pCURE2 inserted as BglII-EcoRI fragment; Cm^R	This work
pLAZ_aFIB	pLAZ2 with anti-FIB segment from pCURE2 inserted as BglII-EcoRI fragment; Cm^R	This work
pLAZ_0-FIB	pLAZ2 with FIB replicon from pO157 plasmid inserted as a BglII-EcoRI fragment; Cm^R	This work
pLAZ_F-FIB	pLAZ2 with FIB replicon from F plasmid inserted as a BglII-EcoRI fragment; Cm^R	This work
pLAZ_F-FIB I234V	pLAZ_FFIB with I234L mutation; Cm^R	This work
pLAZ_ F-FIB F281L	pLAZ_FFIB with F281L mutation; Cm^R	This work
pLAZ_F-FIB I234V_F281L	pLAZ_FFIB with I234L and F281L mutations; Cm^R	This work

pLAZ_F-FIA	pLAZ2 with FIA replicon from F plasmid inserted as a PacIEcoRI fragment; Cm^R	This work
pLAZ_O-FII	pLAZ2 with FII replicon from pO157 plasmid inserted as a BglII-EcoRI fragment; Cm^R	This work
pLAZFIB HybridA	pLAZ2_F/O_Hybrid_FIB spliced at Oligo SOE_F_A_R	This work; Fig.3
pLAZFIB HybridB	pLAZ2_F/O_Hybrid_FIB spliced at Oligo SOE_F_B_R	This work; Fig.3
pLAZFIB HybridC	pLAZ2_F/O_Hybrid_FIB spliced at Oligo SOE_F_C_R	This work; Fig.3
pLAZFIB HybridD	pLAZ2_F/O_Hybrid_FIB spliced at Oligo SOE_F_D_R	This work; Fig.3
pLAZFIB HybridE	pLAZ2_O/F_Hybrid_FIB spliced at Oligo SOE_F_A_R	This work; Fig.3
pLAZFIB HybridF	pLAZ2_O/F_Hybrid_FIB spliced at Oligo SOE_F_B_R	This work; Fig.3
pLAZFIB HybridG	pLAZ2_O/F_Hybrid_FIB spliced at Oligo SOE_F_C_R	This work; Fig.3
pLAZFIB HybridH	pLAZ2_O/F_Hybrid_FIB spliced at Oligo SOE_F_D_R	This work; Fig.3
pLAZ_FFIB_ATG	pLAZ2_FFIB with mutant ATG *rep* start codon	This work
pLAZ_FFIB_GTG	pLAZ2_FFIB with mutant GTG *rep* start codon	This work
pLAZ_FFIB_TTG	pLAZ2_FFIB with mutant TTG *rep* start codon	This work
pLAZ_FFIB_CTC	pLAZ2_FFIB with mutant CTC *rep* start codon	This work
pLAZ_FFIB_CAT	pLAZ2_FFIB with mutant GTG *rep* start codon and C>T suppressor mutation upstream	This work
pLAZ_0FIB_ATG	pLAZ2_0FIB with mutant ATG *rep* start codon	This work
pLAZ_0FIB_GTG	pLAZ2_0FIB with mutant GTG *rep* start codon	This work
pLAZ_0FIB_TTG	pLAZ2_0FIB with mutant TTG *rep* start codon	This work
pLAZ_0FIB_CTC	pLAZ2_0FIB with mutant CTC *rep* start codon	This work
pO157-FIBcat	pLAZ2_0FIB with P15A replicon deleted (NheI-XbaI)	This work

pF-FIBcat	pLAZ2_FFIB with P15A replicon deleted (NheI-XbaI)	This work
pF-mutFIBaph	KLD-Circularised FIB-aph SOE product	This work
pF-FIB.FIIaph	EcoRI-SalI FII from pLAZ_O-FII+EcoRI-SalI WTFIB-aph	This work
pFmutFIB.FIIaph	EcoRI-SalI FII from pLAZ_O-FII+EcoRI-SalI mutFIB-aph	This work
pF-FIB.FIIcat	EcoRI-NheI FII from pLAZ_O-FII + EcoRI-NheI FIB	This work
pFmutFIB.FIIcat	EcoRI-NheI FII from pLAZ_O-FII + EcoRI-NheI mutFIB	This work
pLAZ_F-FIB_egfp	pLAZ2 with F-FIB replicon and *egfp* gene cloned between *rep* gene and downstream iterons; Cm^R	This work
pCT549-i10	Mini IncP-1α, $Kan^R Tet^R$. oriV region defined by HaeII site following iterons 1 to 9.	Thomas & Hussain 1984
pCT549 +i10	Mini IncP-1α, $Kan^R Tet^R$. oriV region engineered to include iterons 1 to 10.	Lazdins et al., 2020
pCT549 +i10+aF	Mini IncP-1α with anti-F cassette inserted, Tet^R	Lazdins et al., 2020
pCT549 -i10+aF	Mini IncP-1α with anti-F cassette inserted, Tet^R	Lazdins et al., 2020
pCT549 +i10+aF ΔFIBrep	Mini IncP-1α with anti-F cassette inserted with FIB *rep* gene inactivated by an in-frame deletion	This work
pCT549 +i10+AF Stop120	Mini IncP-1α with anti-F cassette inserted and a stop codon at position 120 in the *rep* gene of FIB replicon	This work
pCT549 +i10+AF Stop159	Mini IncP-1α with anti-F cassette inserted and a stop codon at position 159 in the *rep* gene of FIB replicon	This work
pCT549 +i10+AF BCD	Mini IncP-1α with anti-F cassette inserted but carrying only the upstream BCDD'D'' repeat region of the FIB replicon	This work

pCT549 +i10+AF EFGHIJ	Mini IncP-1α with anti-F cassette inserted but carrying only the downstream EFGHIJ repeats region of the FIB replicon	This work

a. Plasmid Taxonomic Units from Redondo-Salvo et al., 2021.

Burland, V., Shao, Y., Perna, N.T., Plunkett, G., Sofia, H.J. and Blattner, F.R. (1998) The complete DNA sequence and analysis of the large virulence plasmid of *Escherichia coli* O157: H7. Nucleic Acids Res. **26**, 4196- 4204.

Chang, A.C.Y. and Cohen, S.N. (1978) Construction and characterisation of amplifiable multicopy DNA cloning vehicles derived from P15A cryptic mini-plasmids. J Bacteriol **134**, 1141-1156.

Cottell, J.L., Saw, H.T.H., Webber, M.A. and Piddock, L.J.V. (2014) Functional genomics to identify the factors contributing to successful persistence and global spread of an antibiotic resistance plasmid BMC Microbiology **14**, e168.

Hale, L., Lazos, O., Haines, A.S. and Thomas, C.M. (2010) An efficient stress-free strategy to displace stable bacterial plasmids. BioTechniques, **48**, 223–228.

Lazdins, A., Maurya, A.P., Miller C.E., Kamruzzaman M., Liu, S., Stephens E.R., Lloyd, G., Haratianfar, M., Haines, A.S., Jan-Ulrich Kreft, J.-U., Webber, M.A., Iredell J. and Thomas, C.M. (2020) Potentiation of curing by a broad-host-range self-transmissible vector for displacing resistance plasmids to tackle AMR. PloS ONE **15**, e0225202.

Makino, K., Ishii, K., Yasunaga, T., Hattori, M., Yokoyama, K., Yutsudo, C.H., Kubota, T., Yamaichi, Y., et al. (1998). Complete nucleotide sequences of 93-kb and 3.3-kb plasmids of an enterohemorrhagic Escherichia coli O157:H7 derived from Sakai Outbreak. DNA Res. **5**, 1-9.

Santiago Redondo-Salvo, S., Bartomeus-Peñalver, R., Vielva, L., Tagg, K.A., Webb, H.E., Fernández-López, R. and de la Cruz, F. (2021) COPLA, a taxonomic classifier of plasmids. BMC Bioinformatics 22:390 https://doi.org/10.1186/s12859-021-04299-x

Thomas, C.M. (1981) Complementation analysis of replication and maintenance functions of broad host plasmids RK2 and RP1. Plasmid **5**, 277-291.

Thomas, C.M., and Hussain, A.A.K. (1984) The *korB* gene of broad host range plasmid RK2 is a major copy number control element which may act together with *trfB* by limiting *trfA* expression. EMBO J. **3**, 1513-1519.

Yanisch-Perron, C., Vieira, J. and Messing, J. (1985) Improved M13 phage cloning vectors and host strains: nucleotide sequences of the 13mp18 and pUC19 vectors. Gene **33**, 103-119.

Supplementary Table S2. Oligonucleotides used during this work.

Primer	Sequence (all sequences are written 5'-3')[a]	Template
Amplification of antiF-cassette regions		
aFIB_F	GCTAGAATTCTAGAGGAGGCTCGATCCAGTAAAC	pCURE2
aFIB_R	GCTAAGATCTCAAGCTGTGGTCAGCAGAACAG	
aFIA_F	GCTAGAATTCTCGAGCGGATAGCCAATTCAG	
aFIA_R	GCTAAGATCTGGATCCGCCAGACGACTCATAT	
aFIC_F	GCTAGAATTCCCTGTCGGTAATGAGTGAACAG	
aFIC_R	GCTAAGATCTCGAGGGAACGGACTGGAAAC	
aFIIA_F	GCTAGAATTCGACTGAAGATCAGTCACACCATCC	
aFIIA_R	GCTAAGATCTGGGATAACAGGGTAATCCCGGA	
Site-directed mutagenesis of anti-FIB segment		
Stop 120_F	AGCCAGTCATGACGCAGGCTG	
Stop 120_R	TGAAGGTATACCACACAGTTTTGC	
Stop 159_F	CTGGTACAGTGAGCCTACTAC	
Stop 159_R	ATGGGTGATGTATTCCCG	
Del_F	GGCATCACGCTGGATGAC	
Del_R	ATTGTTTGGTGTCAGTGTCAC	
Site-directed mutagenesis of FIB iterons		
BCD_F	TGACAATTGTCGAGCGGATAGCCAATT	
BCD_R	GTCCTCGAGATTGTTTGGTGTCAGTGTC	
EJ_F	TGACAATTGGGCATCACGCTGGATGAC	
EJ_R	GTCCTCGAGCTGGATCGAGCCTCCTCTA	
Amplification of FIB replicon from F'prolac and pO157		
F_FIB	GCTAGAATTCGTGCCTGTCATCAGGATATTCATG	F'prolac
O157_FIB	GCTAGAATTCGTTCCCGTTTCTGTCTGACGTG	pO157
Site-directed mutagenesis of F FIB replicon		
I234V_F	TCCGGCACCGGTATCGCTGGC	pLAZ_F-FIB

I234V_R	TCCCGGGGCAGGCTCTCTATAAAG	
F281L_F	GCGGACAAAACTCTTCTGTATTCACTAC	
F281L_R	CCCCGCTGGATCTCCGTG	
Site-directed mutagenesis of F and O FIB replicons		
F_ATG_F	GGCTTTATGGAAAACGAAAATTCAG	F'prolac
F_ATG_R	ATTTTCGTTTTCCATAAAGCCTTCTG	
F_GTG_F	GGCTTTGTGGAAAACGAAAATTCAG	
F_GTG_R	ATTTTCGTTTTCCACAAAGCCTTCTG	
F_TTG_F	GGCTTTTTGGAAAACGAAAATTCAG	
F_TTG_R	ATTTTCGTTTTCCAAAAAGCCTTCTG	
F_CTC_F	GGCTTTCTCGAAAACGAAAATTCAG	
F_CTC_R	ATTTTCGTTTTCGAGAAAGCCTTCTG	
F_CAT_F	ACTGTCCATAGGCAGAAGGCTTTG	pLAZ_FFIB_GTG
F_CAT_R	AGCCTTCTGCCTATGGACAGTAAAAG	
O_ATG_R	ATTTTCGTTTTCCATAAAGCCTCCTG	pO157
O_GTG_R	ATTTTCGTTTTCCACAAAGCCTCCTG	
O_TTG_R	ATTTTCGTTTTCCAAAAAGCCTCCTG	
O_CTC_R	ATTTTCGTTTTCGAGAAAGCCTCCTG	
FIB_Sal_R	ATCGGTCGACCAAAGGGTTC	F'prolac and pO157
Amplification of FIA replicon from F'prolac		
F_Full_FIA_F	GCTAGAATTCGCGGATAGCCAATTCAGAG	F'prolac
F_Full_FIA_R	GATCTTAATTAAGAATAAATGCCTTGGCCTTTATATG	
Amplification of FII replicon from pO157		
O157_FII_F	ACACAGATCTTCGTCACAATTCTCAAG	pO157
O157_FII_R	GATCGAATTCTCTGTCCACAGGACAGGCAGTG	
Hybrid FIB replicons from F'prolac and pO157		
SOE_F_A_R	CCATAGTTTATGTCATTAACTTACTTCAG	F'prolac
SOE_F_B_R	CTGACTGAAGAAAAACCTGCAG	

SOE_F_C_R	CCTGTGGACAGTAAAAGAATAAACAG	
SOE_F_D_R	GATGTCTGAATTTTCGTTTTCCAG	
SOE_O_A_F	CTGAAGTAAGTTAATGACATAAACTATGG	pO157
SOE_O_B_F	CTGCAGGTTTTTCTTCAGTCAG	
SOE_O_C_F	CTGTTTATTCTTTTACTGTCCACAGG	
SOE_O_D_F	CTGGAAAACGAAAATTCAGACATC	
Amplification of egfp from pCT::egfp and insertion downstream of FIB rep		
SOE_GFP_F	cccgctgaACACAGGAAACAGCTATGGTGAGCAAGGGCGAGG	pCT::egfp
SOE_GFP_R	ctacagtttatgtCTAAGCTGCTAAAGCGTAGTTTTCGTCGTTTGCTGCCTTGTACAGCTCGTCCATGCCG	
SOE_FIB_F	GCTTTAGCAGCTTAGACATAAACTGTAGTCAGTGAAGAGTGCTC	pLAZ_F-FIB
SOE_FIB_R	CTGTTTCCTGTGTTCAGCGGGATTTGAAGAG	F'prolac and p0157
SOE_FIBd_F	GCTTTAGCAGCTTAGACATAAACTGTAGTCAGTGAAGAGTGCTC	F'prolac
FIBd_R_Sal	ATCGGTCGACCAAAGGGTTC	F'prolac
Dual FIB-FII replicon plasmids		
RBS Mut^n F	CTGTCCACAGGCAGGAGGCTTTCTGGAAAAC	JM109 boilate
RBS Mut^n R	GTTTTCCAGAAAGCCTCCTGCCTGTGGACAG	
FIB end NheI	CTGGCTAGCTAAGCTGTGGTCAGCAGAACAG	
FIBaph SOE	TGGCTTTGTTGAGTGACGGCATACGCGCTTC	
aphFIB SOE	GCCGTCACTCAACAAAGCCACGTTGTGTCTC	pCT549
aph end SalI	CATGTCGACACAGCGTAATGCTCTGCCAGTG	

a. Critical restrictions sites are underlined where they exist.

Supplementary Table S3. Summary of the ability of hybrid and mutant FIB replicons to replicate in *E. coli* C2110.

Replicon	Replication?	Comments
pLAZ_F-FIB	No	F-WT
pLAZ_F-FIB I234V	No	One of two SNP between F and pO157 rep FIB
pLAZ_ F-FIB F281L	No	One of two SNP between F and pO157 rep FIB
pLAZ_F-FIB I234V_F281L	No	Both SNPs changed to be like pO157
pLAZ_FFIB_ATG	Yes	Putative start codon improvement
pLAZ_FFIB_GTG	No	Putative start codon improvement
pLAZ_FFIB_TTG	Yes	Putative start codon improvement
pLAZ_FFIB_CTC	No	Putative start codon inactivation
pLAZ_FFIB_GTG and CAT	Yes	Potential G=C pair in hairpin disrupted
pLAZ_OFIB_ATG	Yes	Putative start codon improvement
pLAZ_OFIB_GTG	No	Putative start codon improvement
pLAZ_OFIB_TTG	Yes	Putative start codon improvement
pLAZ_OFIB_CTC	No	Putative start codon inactivation
pLAZ_O-FIB	Yes	pO157-WT

Supplementary Table S4. Comparison of single FIA, FIB and FII replicon plasmids copy number levels relative to (a) pCT549+i10 and (b) pCT549-i10. See Supplementary Figures 5 and 6 for the gels from which this data came.

(a) Relative to pCT549+i10

Replicon	Band	vol	-bkgd	Size kb	vol/kb	F/P	mean	SD	T Test
FIA	pCT549	84790	30137	15.5	1944	0.850	0.829	0.0300	
	FIA	66889	12236	7.4	1654				FIAvFIB
	bkgd	54653							$P=2x10^{-7}$
FIA	pCT549	99721	41338	15.5	2667	0.831			
	FIA	74775	16392	7.4	2215				FIAvFII
	bkgd	58383							P=0.068
FIA	pCT549	97982	37335	15.5	2409	0.812			
	FIA	75121	14474	7.4	1956				FIBvFII
	bkgd	60647							$P=9.5x10^{-5}$
FIA	pCT549	100869	37593	15.5	2425	0.863			
	FIA	78769	15493	7.4	2094				
	bkgd	63276							
FIA	pCT549	100902	36263	15.5	2340	0.779			
	FIA	78130	13491	7.4	1823				
	bkgd	64639							
FIA	pCT549	99788	35358	15.5	2281	0.839			
	FIA	78600	14170	7.4	1915				
	bkgd	64430							
FIB	pCT549	108448	44153	15.5	2849	1.133	1.132	0.0565	
	FIB	88500	24205	7.5	3227				
	bkgd	64295							
FIB	pCT549	97234	35615	15.5	2298	1.099			
	FIB	80564	18945	7.5	2526				
	bkgd	61619							
FIB	pCT549	96478	35360	15.5	2281	1.042			
	FIB	78938	17820	7.5	2376				
	bkgd	61118							
FIB	pCT549	95575	34588	15.5	2231	1.206			
	FIB	81173	20186	7.5	2691				
	bkgd	60987							
FIB	pCT549	90542	31699	15.5	2045	1.150			
	FIB	76476	17633	7.5	2351				
	bkgd	58843							
FIB	pCT549	87429	31295	15.5	2019	1.162			
	FIB	73735	17601	7.5	2347				
	bkgd	56134							
FII	pCT549	77618	31545	15.5	2035	0.790	0.777	0.0237	
	FII	58932	12859	8	1607				
	bkgd	46073							
FII	pCT549	79463	32537	15.5	2099	0.737			
	FII	59303	12377	8	1547				
	bkgd	46926							
FII	pCT549	79411	32147	15.5	2074	0.768			
	FII	59998	12734	8	1592				
	bkgd	47264							
FII	pCT549	76121	28784	15.5	1857	0.804			
	FII	59280	11943	8	1493				
	bkgd	47337							
FII	pCT549	76335	29344	15.5	1893	0.791			

	FII	58974	11983	8	1498				
	bkgd	46991							
FII	pCT549	76023	29911	15.5	1930	0.771			
	FII	58010	11898	8	1487				
	bkgd	46112							

(b) Relative to pCT549-i10

Replicon	Band	vol	-bkgd	Size (kb)	vol/kb	F/P	mean	SD	T Test
FIA	pCT549	71554	29557	15.5	1907	0.428	0.492	0.0427	
	FIA	48040	6043	7.4	817				FIAvFIB
	bkgd	41997							$P=2.6x10^{-7}$
FIA	pCT549	70395	28858	15.5	1862	0.476			
	FIA	48099	6562	7.4	887				FIAvFII
	bkgd	41537							P=0.169
FIA	pCT549	65636	25259	15.5	1630	0.545			
	FIA	46949	6572	7.4	888				FIBvFII
	bkgd	40377							$P=2.7x10^{-6}$
FIA	pCT549	82745	39903	15.5	2574	0.470			
	FIA	51782	8940	7.4	1208				
	bkgd	42842							
FIA	pCT549	73367	30119	15.5	1943	0.527			
	FIA	50824	7576	7.4	1024				
	bkgd	43248							
FIA	pCT549	80789	36621	15.5	2363	0.508			
	FIA	53057	8889	7.4	1201				
	bkgd	44168							
FIB	pCT549	75784	31090	15.5	2006	0.837	0.775	0.0442	
	FIB	57288	12594	7.5	1679				
	bkgd	44694							
FIB	pCT549	82469	37642	15.5	2429	0.758			
	FIB	58628	13801	7.5	1840				
	bkgd	44827							
FIB	pCT549	82289	37657	15.5	2429	0.796			
	FIB	59135	14503	7.5	1934				
	bkgd	44632							
FIB	pCT549	75540	31636	15.5	2041	0.791			
	FIB	56012	12108	7.5	1614				
	bkgd	43904							
FIB	pCT549	79118	36499	15.5	2355	0.707			
	FIB	55100	12481	7.5	1664				
	bkgd	42619							
FIB	pCT549	73574	32872	15.5	2121	0.760			
	FIB	52788	12086	7.5	1611				
	bkgd	40702							
FII	pCT549	69660	27400	15.5	1768	0.476	0.456	0.0780	
	FII	48992	6732	8	842				
	bkgd	42260							
FII	pCT549	72325	29169	15.5	1882	0.537			
	FII	51240	8084	8	1011				
	bkgd	43156							
FII	pCT549	75902	32989	15.5	2128	0.516			
	FII	51698	8785	8	1098				
	bkgd	42913							
FII	pCT549	77013	34264	15.5	2211	0.408			

	FII	49956	7207	8	901				
	bkgd	42749							
FII	pCT549	63545	22540	15.5	1454	0.325			
	FII	44784	3779	8	472				
	bkgd	41005							
FII	pCT549	67861	26303	15.5	1697	0.473			
	FII	47983	6425	8	803				
	bkgd	41558							

Supplementary Table S5. Displacement and incompatibility of F-FIB/FII dual replicon plasmids.

Incoming plasmid	Target plasmid	Cfu with plas1 10^{-6}	Cfu with plas1+2 10^{-5}	Prop^{n} with both plasmids	Mean	SD	TTEST p=
WT cat#7	WTaph1	12	15	0.125	0.0747	0.0393	0.3799
WT cat#7	WTaph2	28	10	0.0357			
WT cat#8	WTaph1	20	17	0.085			
WT cat#8	WTaph2	17	9	0.0529			
WT cat#7	MUTaph3	15	12	0.08	0.0657	0.0401	
WT cat#7	MUTaph4	20	12	0.06			
WT cat#8	MUTaph3	22	3	0.0136			
WT cat#8	MUTaph4	11	12	0.109			
WTaph#1	WTcat7	15	12	0.08	0.0359	0.031	0.1137
WTaph#1	WTcat8	29	8	0.0276			
WTaph#2	WTcat7	27	2	0.0074			
WTaph#2	WTcat8	28	8	0.0286			
WTaph#1	MUTcat10	29	18	0.0621	0.0598	0.0129	
WTaph#1	MUTcat12	16	9	0.0563			
WTaph#2	MUTcat10	29	13	0.0448			
WTaph#2	MUTcat12	25	19	0.076			

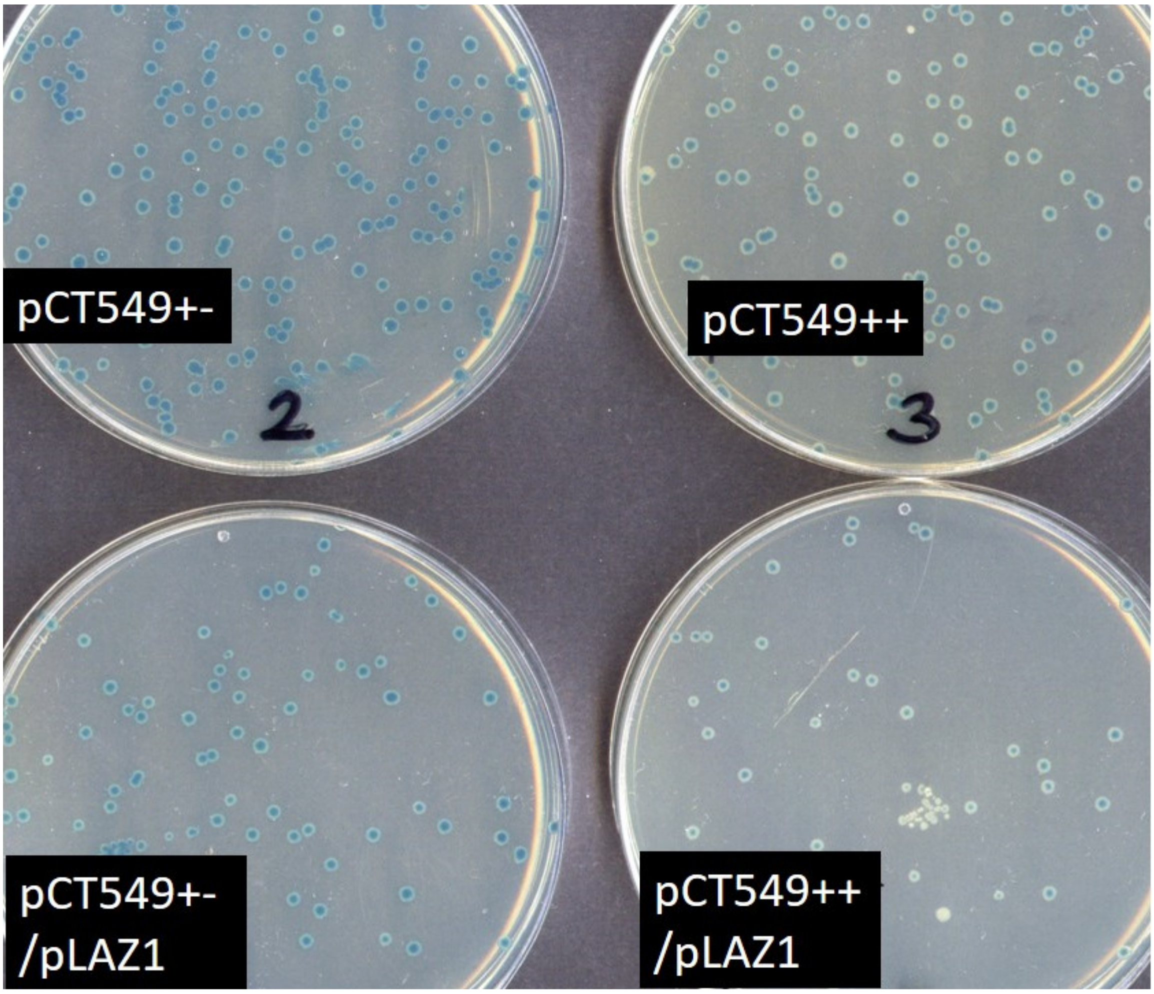

Supplementary Figure S1. Tet-Amp-IPTG-Xgal (upper pair) and Tet-Amp-Cam-IPTG-Xgal (lower pair) agar plates showing the phenotype of JM109(pUC18) transformed with pCT549+iteron10 without(-) and with (+) the anti-F cassette, alone or co-transformed with pLAZ1. Key points to note are: although almost all colonies with pCT549++ are blue, they are paler and have a white rim, indicating consistent depression of F'prolac copy number and segregation; the slightly paler blue on the double transformation plates; the very clearly white colony on the pCT549++/pLAZ1 plate.

Alignment statistics for match

```
F     38,021 GTGTATGCTGTTTTTCCGGAATGATTTATCCACATATCCAGAGGCCCGATCCAGTAAATA  38,080
             |||||||||||||  | |||||||||||||||||||||| ||||| |||||||||||| |
pO157 26,878 GTGTATGCTGTTTCCCTGGAATGATTTATCCACATATCCGGAGGCTCGATCCAGTAAACA  26,937

F     38,081 GATCCATGAATGATCAATAAAGGATCCATTAAAGATCCCCATATCGCTACAAACCTTGTC  38,140
             ||||||||||||||||| ||||||||||||||||||||||||| |||| |||||||||||
pO157 26,938 GATCCATGAATGATCAACAAAGGATCCATTAAAGATCCCCATACCGCTGCAAACCTTGTC  26,997
                                    B                    C
F     38,141 ACTCATGGGCCGGGACCACGTTCACATAAGCTGTGGCATGTTATGGATAAACTGTAACAG  38,200
             |||||||||||||||||||| |||||||||| |||||||||||  ||||||||||||||
pO157 26,998 ACTCATGGGCCGGGACCACGATCACATAAGCAGTGGCATGTTACTGATAAACTGTAACAT  27,057

                  D                       D′                    D*
F     38,201 GTTCATGATAAGCTGTATTCAGTAATGCATATACTGAAGTAAGTTAATGACATAAACTAT  38,260
             | | |||||||||||||||||||||| |||||||||||||||||||||||||||||||||
pO157 27,058 GCTAATGATAAGCTGTATTCAGTAATCCATATACTGAAGTAAGTTAATGACATAAACTAT  27,117

F     38,261 GGTCAGTACGCCAGACTCAGTTGTTAAATACAGACTGCAGGTTTTTCTTCAGTCAGTTGA  38,320
             |||||||||||||||||||| |||||||||||| ||||||||||||||||||||||||
pO157 27,118 GGTCAGTACGCCAGACTCAGCTGTTAAATACAGGCTGCAGGTTTTTCTTCAGTCAGTTAG  27,177

F     38,321 CGGGGTTCTGACACACGATTTTCTGTTTATTCTTTTACTGTCCACAGGCAGAAGGCTTTC  38,380
             ||||| ||||||||||||||| ||||||||||||||||||||||||||||| ||||||||
pO157 27,178 CGGGGCTCTGACACACGATTTGCTGTTTATTCTTTTACTGTCCACAGGCAGGAGGCTTTC  27,237
                                                               RBS

F     38,381 TGGAAAACGAAAATTCAGACATCAAAAAACTGTTCGGCGAGGTGGATAAGTCGTCCGGTG  38,440
             ||||||||||||||||||||||||||||||||||||||||||||||||||||||||||||
pO157 27,238 TGGAAAACGAAAATTCAGACATCAAAAAACTGTTCGGCGAGGTGGATAAGTCGTCCGGTG  27,297
             M  E  N  E  N  S  D  I  K  K  L  F  G  E  V  D  K  S  S  G 20

F     38,441 AGCTGGTGACACTGACACCAAACAATAACAACACCGTACAACCTGTGGCGCTGATGCGTC  38,500
             ||||||||||||||||||||||||||||||||||||||||||||||||||||||||||||
pO157 27,298 AGCTGGTGACACTGACACCAAACAATAACAACACCGTACAACCTGTGGCGCTGATGCGTC  27,357
             E  L  V  T  L  T  P  N  N  N  N  T  V  Q  P  V  A  L  M  R 40

F     38,501 TGGGCGTTTTTGTACCGACCCTTAAATCACTGAAGAACAGTAAAAAAAATACACTGTCAC  38,560
             ||||||||||||||||||||||||||||||||||||||||||||||||||||||||||||
pO157 27,358 TGGGCGTTTTTGTACCGACCCTTAAATCACTGAAGAACAGTAAAAAAAATACACTGTCAC  27,417
             L  G  V  F  V  P  T  L  K  S  L  K  N  S  K  K  N  T  L  S 60

F     38,561 GTACTGATGCCACGGAAGAGCTGACTCGTCTTTCCCTGGCCCGTGCTGAGGGATTCGATA  38,620
             ||||||||||||||||||||||||| ||||||||||||||||||||||||||||||||||
pO157 27,418 GTACTGATGCCACGGAAGAGCTGACACGTCTTTCCCTGGCCCGTGCTGAGGGATTCGATA  27,477
             R  T  D  A  T  E  E  L  T  R  L  S  L  A  R  A  E  G  F  D 80

F     38,621 AGGTTGAGATCACCGGCCCCCGCCTGGATATGGATAACGATTTCAAGACCTGGGTGGGGA  38,680
             ||||||||||||||||||||||||||||||||||||||||||||||||||||||||||||
pO157 27,478 AGGTTGAGATCACCGGCCCCCGCCTGGATATGGATAACGATTTCAAGACCTGGGTGGGGA  27,537
             K  V  E  I  T  G  P  R  L  D  M  D  N  D  F  K  T  W  V  G 100

F     38,680 TCATTCATTCCTTTGCCCGCCATAACGTGATTGGTGACAAAGTTGAACTGCCTTTTGTTG  38,740
             |||||||||||||||||||||||||||| ||||||||||||||||||||||||||||| |
pO157 27,538 TCATTCATTCCTTTGCCCGCCATAACGTAATTGGTGACAAAGTTGAACTGCCTTTTGTCG  27,597
             I  I  H  S  F  A  R  H  N  V  I  G  D  K  V  E  L  P  F  V 120

F     38,741 AGTTTGCAAAACTGTGTGGTATACCTTCAAGCCAGTCATCCCGCAGGCTGCGTGAGCGCA  38,800
             |||||||||||||||||||||||||||||||||||||||| |||||||||||||||||||
pO157 27,598 AGTTTGCAAAACTGTGTGGTATACCTTCAAGCCAGTCATCACGCAGGCTGCGTGAGCGCA  27,657
             E  F  A  K  L  C  G  I  P  S  S  Q  S  S  R  R  L  R  E  R 140

F     38,801 TCAGCCCTTCCCTGAAGCGCATTGCCGGTACCGTGATCTCGTTTTCCCGTACCGATGAGA  38,860
             |||||||||||||||||||||||||||||||||||||||| |||||||||||||||||||
pO157 27,658 TCAGCCCTTCCCTGAAACGCATTGCCGGTACCGTGATCTCCTTTTCCCGCACCGATGAGA  27,717
             I  S  P  S  L  K  R  I  A  G  T  V  I  S  F  S  R  T  D  E 160

F     38,861 AGCACACCCGGGAATACATCACCCATCTGGTACAGTCAGCCTACTACGATACTGAGCGGG  38,920
             ||||||||||||||||||||||||||||||||||||||||||||||||||||||| ||||
pO157 27,718 AGCACACCCGGGAATACATCACCCATCTGGTACAGTCAGCCTACTACGATACTGAACGGG  27,777
             K  H  T  R  E  Y  I  T  H  L  V  Q  S  A  Y  Y  D  T  E  R 180
```

```
F      38,921 ATATTGTTCAGTTACAGGCTGATCCCCGTCTGTTTGAACTGTACCAGTTTGACAGAAAAG  38,980
              |||||||||||||||||||||||||||| || |||||||||||||||||||||||||| |
pO157 27,778 ATATTGTTCAGTTACAGGCTGATCCCCGCCTTTTTGAACTGTACCAGTTTGACAGAAAGG  27,837
              D  I  V  Q  L  Q  A  D  P  R  L  F  E  L  Y  Q  F  D  R  K 200

F      38,981 TCCTTCTCCAGCTTAAGGCGATTAATGCCCTGAAGCGACGGGAGTCCGCCCAGGCACTCT  39,040
              ||||||||||||||||||||||||||||||||||||||||||||||||||||||||||||
pO157 27,838 TCCTTCTCCAGCTTAAGGCGATTAATGCCCTGAAGCGACGGGAGTCCGCCCAGGCACTCT  27,897
              V  L  L  Q  L  K  A  I  N  A  L  K  R  R  E  S  A  Q  A  L 220

F      39,041 ACACCTTTATAGAGAGCCTGCCCCGGGATCCGGCACCGATATCGCTGGCGCGGCTGCGTG  39,100
              |||||||||||||||||||||||||||||||||||||| |||||||||||||||||||||
pO157 27,898 ACACCTTTATAGAGAGCCTGCCCCGGGATCCGGCACCGGTATCGCTGGCGCGGCTGCGTG  27,957
              Y  T  F  I  E  S  L  P  R  D  P  A  P V>I S  L  A  R  L  R 240

F      39,101 CACGCCTCAATCTGAAGTCTCCTGTATTTTCCCAGAACCAGACGGTCAGACGGGCAATGG  39,160
              ||||||||||||||||||||||||||||||||||||||||||||||||||||||||||||
pO157 27,958 CACGCCTCAATCTGAAGTCTCCTGTATTTTCCCAGAACCAGACGGTCAGACGGGCAATGG  28,017
              A  R  L  N  L  K  S  P  V  F  S  Q  N  Q  T  V  R  R  A  M 260

F      39,161 AGCAGTTGCGCGAGATTGGATATCTTGATTACACGGAGATCCAGCGGGGGCGGACAAAAT  39,220
              ||||| |||||||||||||||||||||||||||||||||||||||||||||||||||||
pO157 28,018 AGCAGCTGCGCGAGATTGGATATCTTGATTACACGGAGATCCAGCGGGGGCGGACAAAAC  28,077
              E  Q  L  R  E  I  G  Y  L  D  Y  T  E  I  Q  R  G  R  T  K 280

F      39,221 TCTTCTGTATTCACTACCGGCGTCCCCGGTTAAAAGCGCCGAATGATGAGAGTAAGGAAA  39,280
              ||||||| ||||||||||||||||||||||||||||| ||||||||||||||||||||||
pO157 28,078 TCTTCTGCATTCACTACCGGCGTCCCCGGTTAAAAGCACCGAATGATGAGAGTAAGGAAA  28,137
            L>F F  C  I  H  Y  R  R  P  R  L  K  A  P  N  D  E  S  K  E 300

F      39,281 ATCCGTTGCCACCTTCACCTGCGGAAAAAGTCAGTCCGGAGATGGCGGAGAAACTTGCCC  39,340
              |||||||||||||||||||||||||||||||||||||||||||||||||||| |||||||
pO157 28,138 ATCCGTTGCCACCTTCACCTGCGGAAAAAGTCAGTCCGGAGATGGCGGAGAAGCTTGCCC  28,197
              N  P  L  P  P  S  P  A  E  K  V  S  P  E  M  A  E  K  L  A 320

F      39,341 TGCTTGAAAAACTGGGCATCACACTGGATGACCTGGAAAAACTCTTCAAATCCCGCTGAA  39,400
              ||||||| |||||||||||||| |||||||||||||||||||||||||||||||||||||
pO157 28,198 TGCTTGAGAAACTGGGCATCACGCTGGATGACCTGGAAAAACTCTTCAAATCCCGCTGAA  28,257
              L  L  E  K  L  G  I  T  L  D  D  L  E  K  L  F  K  S  R 339

              E                                F
F      39,401 CATAAACTGTAGTCAGTGAAGAGTGCTCCTTTACTGACTACAGCTTATATTATCAGGTGC  39,460
              ||||||||||||||||||||||||| ||||||||||||||||||||||||||||||||||
pO157 28,258 CATAAACTGTAGTCAGTGAAGAGTGTTCCTTTACTGACTACAGCTTATATTATCAGGTGC  28,317

                    G
F      39,461 AGTGAGTGGTCTGCTCACTGCTGTTTATATTCAGTTTCCTGCAGTGCTGCCTGTGGCTGA  39,460
              ||||||||||||||||||||| |||||||||||||||||||||||||||||||| |||||
pO157 28,318 AGTGAGTGGTCTGCTCACTGCAGTTTATATTCAGTTTCCTGCAGTGCTGCCTGTAGCTGA  28,377

F      39,461 GCTGCCATCTGCCTGTCCCTTACGTGAGTCACCCCGTAACCTGATGCTGAGGCATTGCTC  39,520
              |||| |||||||| ||||||||||||||||||||||||||||||||||||||||||||||
pO157 28,378 GCTGTCATCTGCCGGTCCCTTACGTGAGTCACCCCGTAACCTGATGCTGAGGCATTGCTC  28,437

                               H                    I
F      39,521 CCTTCATAAAACATGGTTTACTCACTACAGCTTATCTACCTGCTCCAGCTTATGTTATGT  39,580
              |||||||||||||||  |||||||||||||||||| ||| ||||||||||||||||||||
pO157 28,438 CCTTCATAAAACATGACTTACTCACTACAGCTTATATACATGCTCCAGCTTATGTTATGT  28,497

                J
F      39,581 CTGTTCTGCTGACCACAGCTTATATAAGGAAGCGCGTATGCCGTCACTTCAGGGGAGGCA  39,640
              ||||||||||||||||||||||||||||||||||||||||||||||| ||||||||||||
pO157 28,498 CTGTTCTGCTGACCACAGCTTATATAAGGAAGCGCGTATGCCGTCACCTCAGGGGAGGCA  28,557

F      39,641 GTGTACGCAGGATCTCCGCAGCATCCCGACCGTCACCTGTGAAAGGCACTGCCAGCGTGG  39,700
              |||||||||||||||||||||||||||| |||||||||||||||||||||||||||||||
pO157 28,558 GTGTACGCAGGATCTCCGCAGCATCCCGCCCGTCACCTGTGAAAGGCACTGCCAGCGTGG  28,617

F      39,701 CAGCCATATCCAGTGCAAACACTCTGGTATAAACCTCCATCGAACGTGGATCCCTGTGAC  39,760
              ||||||||||||| ||||||||||||||||||||||||||||||||||||||||||||||
pO157 28,618 CAGCCATATCCAGCGCAAACACTCTGGTATAAACCTCCATCGAACGTGGATCCCTGTGAC  28,677

F      39,761 CAGCCAGTGCCTGGATGACTTTCCGGGGCTGGCGGTGATAGAGCATGTGCATGATATAGC  39,820
              | ||||| |||||||||||||||||||||||||||||||||||||| |||||||||||||
pO157 28,678 CTGCCAGGGCCTGGATGACTTTCCGGGGCTGGCGGTGATAGAGCATATGCATGATATAGC  28,737
```

```
F      39,821 TGTGCCGGAAGGTGTGTGGTGTGACCGGAATCGAAAAGTGTACTCCGTCAGCTTCGGCCC  39,880
              ||||||||||||||||||||||||||||||||||||||||||||||||||||||||||||
pO157 28,738 TGTGCCGGAAGGTGTGTGGTGTGACCGGAATCGAAAAGTGTACTCCGTCAGCTTCGGCCC  28,797

F      39,881 GTCTGACAGCCTGCTTCAGCCAGTTGCGCATGGTTTCGTCGGTCACGGCCCATAATGGTT  39,940
              ||||||||||||||||||||||||||||||||||||||||||||||||||||||||||||
pO157 28,798 GTCTGACAGCCTGCTTCAGCCAGTTGCGCATGGTTTCGTCGGTCACGGCCCATAATGGTT  28,857

                                                        K
F      39,941 CACGACGACGGGGCCGGGTGGTGATCATCCAGCTTTCCATCTGCCTGACATAGCTTATAT  40,000
              ||||||||||||||||||||||||||||||||||||||||||||||||||||||||||||
pO157 28,858 CACGACGACGGGGCCGGGTGGTGATCATCCAGCTTTCCATCTGCCTGACATAGCTTATAT  28,917
```

Supplementary Figure S2. Comparison of pO157 and F FIB *rep* genes and flanking regions from Genbank accessions AP018692 and AP001918. The RBS and rep ORF are highlighted in green and the stop codon is highlighted in red. The amino acid differences between the F and pO157 Rep are highlighted in cyan. The Iterons B to D* and E to K are highlighted in yellow.

```
         .         .         .         .         .         60
MENENSDIKKLFGEVDKSSGELVTLTPNNNNTVQPVALMRLGVFVPTLKSLKNSKKNTLS
MENENSDIKKLFGEVDKSSGELVTLTPNNNNTVQPVALMRLGVFVPTLKSLKNSKKNTLS

         .         .         .         .         .        120
RTDATEELTRLSLARAEGFDKVEITGPRLDMDNDFKTWVGIIHSFARHNVIGDKVELPFV
RTDATEELTRLSLARAEGFDKVEITGPRLDMDNDFKTWVGIIHSFARHNVIGDKVELPFV

         .         .         .         .         .        180
EFAKLCGIPSSQSSRRLRERISPSLKRIAGTVISFSRTDEKHTREYITHLVQSAYYDTER
EFAKLCGIPSSQSSRRLRERISPSLKRIAGTVISFSRTDEKHTREYITHLVQSAYYDTER

         .         .         .         .         .        240
DIVQLQADPRLFELYQFDRKVLLQLKAINALKRRESAQALYTFIESLPRDPAPVSLARLR
DIVQLQADPRLFELYQFDRKVLLQLKAINALKRRESAQALYTFIESLPRDPAPISLARLR

         .         .         .         .         .        300
ARLNLKSPVFSQNQTVRRAMEQLREIGYLDYTEIQRGRTKLFCIHYRRPRLKAPNDESKE
ARLNLKSPVFSQNQTVRRAMEQLREIGYLDYTEIQRGRTKFFCIHYRRPRLKAPNDESKE

         .         .         .      339.
NPLPPSPAEKVSPEMAEKLALLEKLGITLDDLEKLFKSR*
NPLPPSPAEKVSPEMAEKLALLEKLGITLDDLEKLFKSR*
```

Supplementary Figure S3. Comparison of F and pO157 FIB Rep proteins.

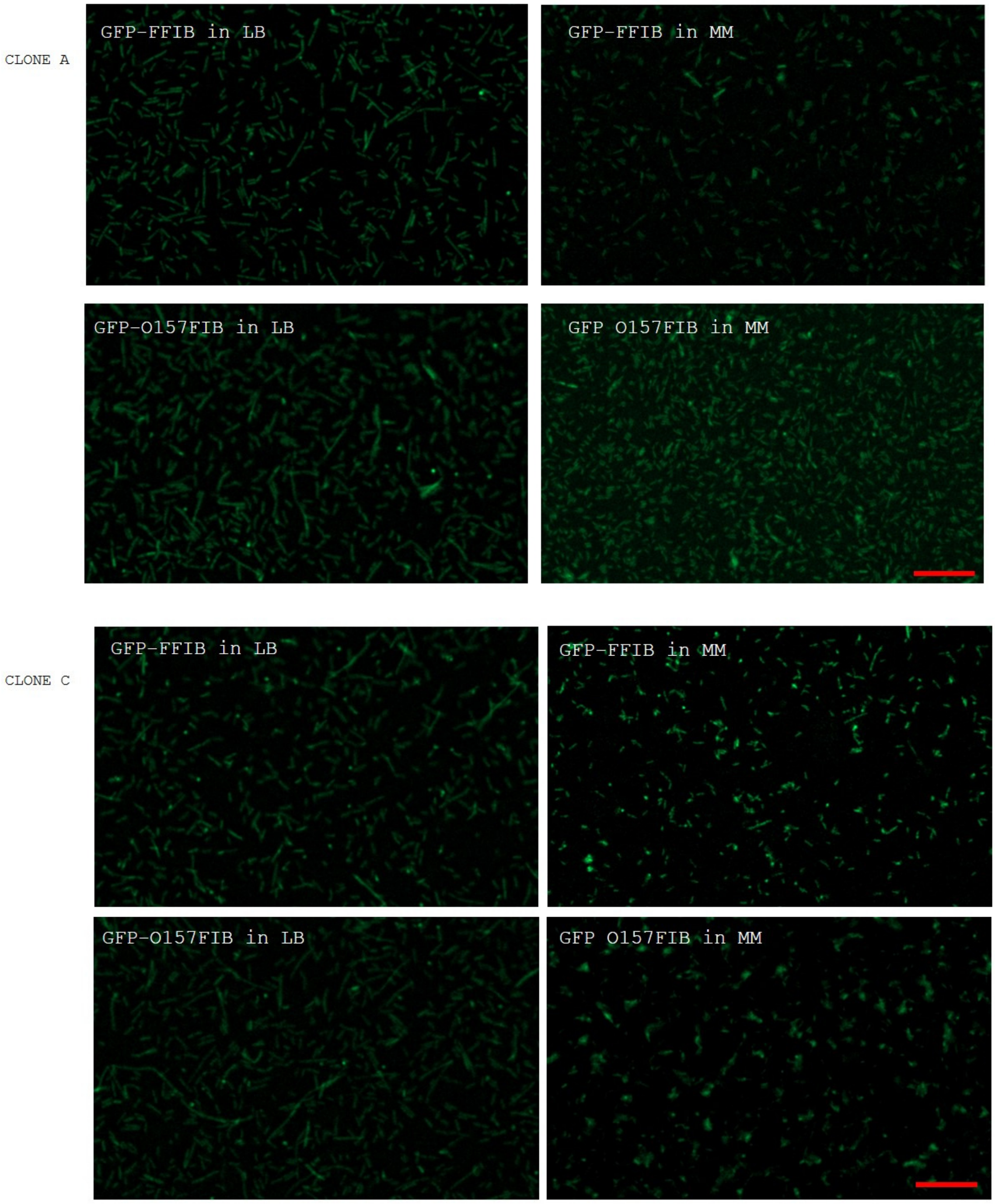

Supplementary Figure S4. Fluorescent widefield microscopy images of *Escherichia coli* strains carrying the plasmid pO157 and F-FIB cultures in either L-broth/L-agar (X', Y') or M9 Minimal Medium (X, Y). Tiled (3x2) Images were acquired with a Zeiss Cell Discoverer 7 equipped with a 20x/0.95 Plan-Apochromat lens. The red scale bar represents 20 µm.

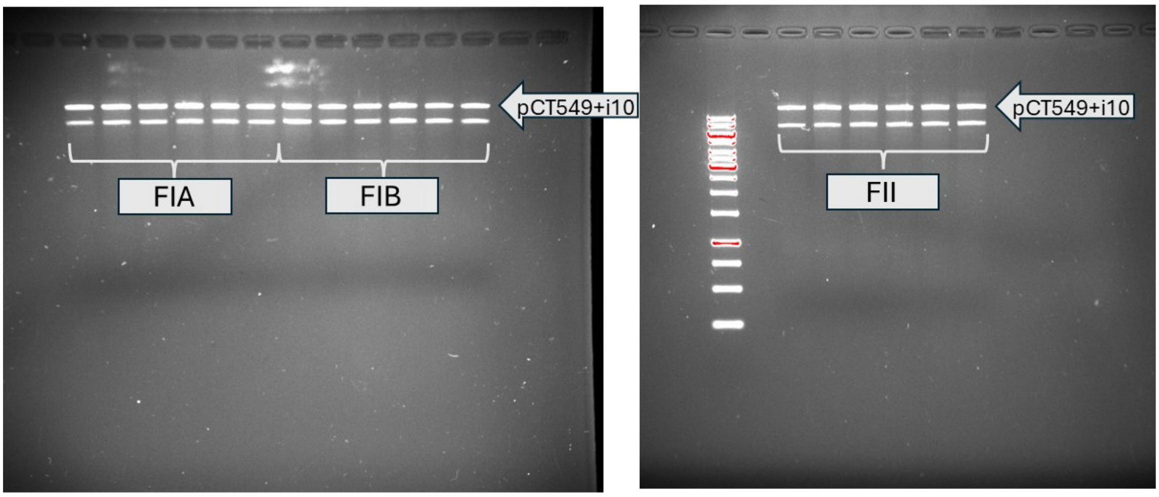

Supplementary Figure S5. Images of the gels from which the data in Table 2 and Supplementary Table S4(a) was generated.

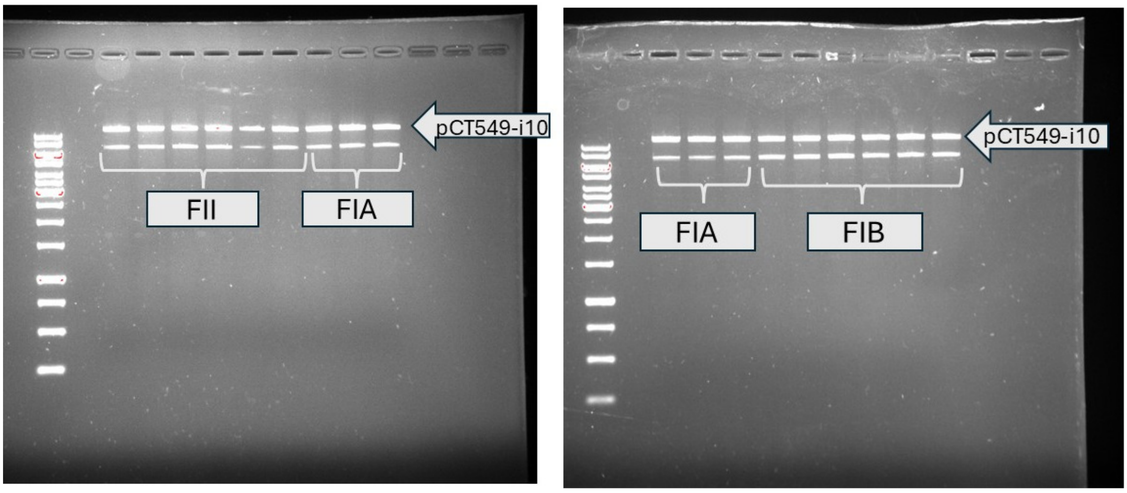

Supplementary Figure S6. Images of the gels from which the data in Table 2 and Supplementary Table S4(b) was generated.
